# Mortality risk score for patients with Chagas cardiomyopathy and pacemaker

**DOI:** 10.1371/journal.pntd.0012114

**Published:** 2024-05-09

**Authors:** Giselle de Lima Peixoto, Sérgio Freitas de Siqueira, Silvana Angelina D’Orio Nishioka, Anísio Alexandre Andrade Pedrosa, Ricardo Alkmim Teixeira, Roberto Costa, Martino Martinelli Filho

**Affiliations:** Heart Institute (InCor), University of São Paulo Medical School, São Paulo, Brazil; Wadsworth Center, UNITED STATES

## Abstract

**Background:**

Prognosis of Chronic Chagasic Cardiomyopathy (CCC) patients depends on functional and clinical factors. Bradyarrhythmia requiring pacemaker is a common complication. Prognosis of these patients is poorly studied, and mortality risk factors are unknown. We aimed to identify predictors of death and to define a risk score for mortality in a large cohort of CCC patients with pacemaker.

**Methods:**

It was an observational, unicentric and prospective study. The endpoint was all-cause mortality. Cox regression was used to identify predictors of death and to define a risk score. Bootstrapping method was used to internal score validation.

**Results:**

We included 555 patients and after a mean follow-up of 3.7±1.5 years, 100 (18%) deaths occurred. Predictors of death were: right ventricular dysfunction (HR [hazard ratio] 2.24; 95%CI 1.41–3.53; *P* = 0.001); heart failure class III or IV (HR 2.16; 95% confidence interval [95%CI] 1.16–4.00; *P* = 0.014); renal disease (HR 2.14; 95%CI 1.24–3.68; *P* = 0.006); left ventricular end-systolic diameter > 44mm (HR 1.97; 95%CI 1.26–3.05; *P* = 0.003); atrial fibrillation (HR 1.94; 95%CI 1.25–2.99; *P* = 0.003) and cardiomegaly on X-ray (HR 1.87; 95%CI 1.10–3.17; *P* = 0.020). The score identified patients with: low (0–20 points), intermediate (21–30 points) and high risk (>31points).

The optimism-corrected C-statistic of the predictive model was 0.751 (95% CI 0.696–0.806). Internal validation with bootstrapping revealed a calibration slope of 0.946 (95% CI 0.920–0.961), reflecting a small degree of over-optimism and C-statistic of 0.746 (95% CI 0.692–0.785).

**Conclusions:**

This study identified predictors of mortality in CCC patients with pacemaker defining a simple, validated and specific risk score.

## Author summary

Chronic Chagas Cardiomyopathy (CCC) is the most important clinical manifestation of chronic Chagas disease. Bradyarrhythmias caused by sinus node disease, atrio and intraventricular block occur in above 50% and requirement of permanent pacing is common. Prognosis of these patients is poorly studied, and mortality risk factors are unknown. Previous studies have identified predictors of death in CCC patients however CCC patients requiring pacemaker were underrepresented. We identified predictors of mortality in CCC patients with pacemaker defining a simple, validated and specific risk score. Predictors of death were: right ventricular dysfunction, heart failure class III or IV, renal disease, left ventricular end-systolic diameter > 44mm, atrial fibrillation and cardiomegaly on X-ray.

## Introduction

Chagas disease is an inflammatory cardiomyopathy caused by the protozoan Trypanosoma cruzi, currently affects 6–8 million people and is responsible for approximately 12,000 deaths per year [1]. Although its incidence has been decreasing, Chagas disease is spreading throughout the world as a consequence of the influx of immigrants from endemic countries and remains as the third largest parasitic disease burden globally [2,3].

Chronic Chagas Cardiomyopathy (CCC) is the most important clinical manifestation of chronic Chagas disease, occuring in 20–40% of infected individuals [4]. Data from infected blood donors in Brazil showed an annual rate of progression to CCC of 1.8% to 5% per year [5].

CCC is a chronic myocarditis that slowly deteriorates the contractile function and the conduction system, it is associated with heart failure, tachyarrhythmias, bradyarrhythmias, thromboembolism, stroke, and death (4). Bradyarrhythmias caused by sinus node disease, atrio and intraventricular block occur in above 50% and requirement of permanent pacing is common, 3.5% to 14.1% of CCC patients [6–8].

Pacemaker use can affect adversely the heart and its impact in CCC patients with and without ventricular dysfunction is unknown [9,10]. Previous studies have identified predictors of death in CCC patients [11,12] however CCC patients requiring pacemaker were underrepresented. In this study we aimed to identify predictors of death and to develop a risk score for mortality in these patients.

## Methods

### Ethics statement

The study protocol was approved by the Local Ethic Committee—Comissão de Ética para Análise de Projetos de Pesquisa do Hospital das Clínicas da Faculdade de Medicina da Universidade de São Paulo (SDC 3608/11/026) All patients signed the informed consent. This prospective cohort study included outpatients with CCC and pacemaker followed at the Heart Institute, Hospital das Clínicas, University of São Paulo. All patients had at least two positive serologic tests for *T*. *cruzi*, pacemaker indication according to Brazilian guidelines [13] and age greater than or equal to 18 years. Patients with associated cardiomyopathies (ischemic, hypertensive or valvular heart disease), cardiac resynchronization therapy (CRT) or implantable cardioverter defibrillator (ICD) were excluded.

Primary endpoint was all-cause mortality. Deaths were classified as cardiovascular, sudden, noncardiovascular or unknown. Patients who underwent heart transplantation and upgraded to CRT or ICD, during follow-up, were excluded, due to the potential confounding effect in the CCC natural history and survival.

At the time of enrollment, all patients underwent a clinical and device assessment, chest x-ray, 12-lead electrocardiography and echocardiography. Clinical and device assessment were performed every 6-months. Clinical evaluation included New York Heart Association (NYHA) HF functional class, history of syncope, comorbidities and medications. A team of experts in cardiac electronic devices performed the pacemaker interrogation enabling the retrieval of the pacing mode, ventricular pacing burden and ventricular arrhythmias. The measurement and reference values for echocardiographic parameters followed the American guidelines recommendations, and the left ventricular ejection fraction (LVEF) was measured by means of the Simpson method. The right ventricle function was subjectively evaluated (good, moderate or poor), and any dysfunction degree was considered right ventricle dysfunction.

The patients were followed for at least 24 months. Throughout the study, clinical approach and therapy management were performed according to the judgment of institutional physicians.

QRS duration was measured in the bipolar II derivation and performed only by the principal investigator. This measurement was performed in pacing beats for pacemaker dependent patients or no-pacing beats in case of sinus node disease. Cardiomegaly, as determined by chest radiography, was defined by a cardiothoracic ratio of more than 0.50.

At any pacemaker interrogation, we considered nonsustained ventricular tachycardia (NSVT) as three or more consecutive premature ventricular complexes with a heart rate of more than 100 beats per minute, lasting less than 30sec. If the episode lasted more than 30sec or was associated with hemodynamic compromise it was classified as sustained ventricular tachycardia (SVT).

Sudden death was defined as natural death preceded by a sudden loss of consciousness within 1 h of the onset of acute symptoms in a previously stable patient.

### Statistical analysis

Categorical and continuous variables were presented as numbers/percentages and means/standard deviations, respectively. All variables were submitted to univariate Cox regression analysis. Continuous variables with *P*<0.20 in the univariate Cox regression analysis were analyzed by the area under de ROC curve to establish the better cutoff value. Categorical variables with *P*<0.20 in the univariate Cox regression analysis were included in the multivariate model using backward selection and *P*<0.05 as a criterion for retaining variables in the model.

To calculate the risk score, the beta coefficient was multiplied by 20 and the result of every variable in the final model was rounded to the nearest integer. Patients were divided into three risk groups.

The discriminative performance of the model was measured using Harrell’s C-statistic. The model was validated using 5.000 bootstrap samples. The degree of optimism was estimated by the average calibration slope of the bootstrap samples.

Survival was estimated by the Kaplan—Meier method, and differences in survival between groups were assessed by the log-rank test.

Two-sided *P* values of less than 0.05 were considered to indicate statistical significance. All data were analyzed by using SPSS 20.0 and software R 3.5.1 (R Foundation, Vienna, Austria).

## Results

### Patient characteristics and follow-up

The cohort analyzed 617 patients included between May 2011 and May 2015, however the final analysis for score development was performed with 555 patients. Twenty-seven patients did not meet the inclusion criteria, two patients refused, 11 were lost during follow-up and 22 underwent device upgrade and one patient received heart transplantation. All the data is available on S1 Data file.

From the 555 patients, the mean age was 63.3±12.0 years, most of them were women (64.0%) and 95% had NYHA I or II (Table 1). The mean LVEF and the QRS duration were 50.5±13.7% and 158.4±32.3msec, respectively. Most of the patients underwent pacemaker implantation due to atrioventricular block and the mean ventricular pacing was 82.3±30.6%. NSVT pacemaker interrogation was retrieved in 30.0%.

**Table 1:** Baseline characteristics and univariate analysis.

	All patients (n = 555)	Survivors (n = 455)	Nonsurvivors (n = 100)	Hazard ratio (95%CI)	*P*
***Clinical and ECG variables***					
Age (years)	63.3±12.0	63.1±11.7	64.4±13.1	1.01 (0.99–1.03)	0.280
Female sex (n/%)	356/64.0	298/65.0	58/58.0	0.74 (0.50–1.10)	0.131
NYHA III/IV (n/%)	27/5.0	15/3.0	12/12.0	3.26 (1.78–5.96)	<0.001
Hypertension (n/%)	400/72.0	330/73.0	70/70.0	0.87 (0.57–1.34)	0.527
Diabetes (n/%)	84/15.0	71/16.0	13/13.0	0.83 (0.47–1.49)	0.540
Dyslipidemia (n/%)	196/35.0	160/35.0	36/36.0	1.02 (0.68–1.53)	0.937
Renal disease (n/%)	39/7.0	23/5.0	16/16.0	2.98 (1.75–5.09)	<0.001
AF (n/%)	108/19.0	70/15.0	38/38.0	2.81 (1.87–4.21)	<0.001
Post-pacemaker syncope (n/%)	67/12.0	49/11.0	18/18.0	1.69 (1.01–2.82)	0.044
QRS duration (msec)	158.4±32.3	155.4±31.5	172.1±32.7	1.02 (1.01–1.02)	<0.001
QRS duration < 165msec (n/%)	215/39.0	160/35.0	55/55.0	2.06 (1.39–3.06)	<0.001
Cardiomegaly on X-ray (n/%)	295/53	216/47	79/79.0	3.68 (2.28–5.96)	<0.001
***Pacemaker related variables***					
Pacemaker time (years)	11.7±9.1	11.5±9.1	13.0±9.1	1.02 (1.00–1.04)	0.130
Pacemaker time > 6years (n/%)	368/66.0	294/65.0	74/74.0	1.48 (0.95–2.31)	0.086
Pacemaker indication (n/%)					
AV Block	412/74.0	340/75.0	72/72.0		
Sinus node disease	117/21.0	99/22.0	18/18.0	0.87 (0.52–1.45)	0.589
Low rate AF	26/5.0	16/4.0	10/10.0	2.42 (1.25–4.69)	0.009
NSVT at pacemaker	167/30.0	130/29.0	37/37.0	1.42 (0.94–2.13)	0.093
VP (%)	82.3±30.6	81.2±31.7	87.3±23.9	1.01 (1.00–1.01)	0.081
VP>40% (n/%)	478/86.0	387/85.0	91/91.0	1.72 (0.86–3.40)	0.123
***Echocardiographic variables***					
LA (mm)	40.7±6.4	39.9±6.2	44.1±6.5	1.09 (1.06–1.12)	<0.001
LA>42mm (n/%)	184/33.0	126/28.0	58/58.0	3.18 (2.14–4.73)	<0.001
LVEF	50.5±13.7	52.3±12.3	42.2±16.4	0.95 (0.94–0.97)	<0.001
LVEF<40% (n/%)	147/26.0	93/20.0	54/54.0	3.82 (2.57–5.66)	<0.001
LVEDD (mm)	53.3±8.0	52.4±7.0	57.3±10.8	1.07 (1.04–1.09)	<0.001
LVEDD>60mm (n/%)	94/17.0	57/13.0	37/37.0	3.33 (2.22–5.00)	<0.001
LVESD (mm)	39.7±9.9	38.4±8.6	45.8±13.0	1.06 (1.04–1.08)	<0.001
LVESD>44mm (n/%)	140/25.0	90/20.0	50/50.0	3.44 (2.32–5.09)	<0.001
RV dysfunction (n/%)	90/16.0	47/10.0	43/43.0	4.69 (3.15–6.97)	<0.001
***Medications***					
ACEi/ARB (n/%)	410/74.0	332/73.0	78/78.0	1.26 (0.79–2.03)	0.334
Beta-blocker (n/%)	323/58.0	246/54.0	77/77.0	2.59 (1.63–4.13)	<0.001
Diuretic agent (n/%)	140/25.0	90/20.0	50/50.0	3.43 (2.32–5.08)	<0.001
Aldosterone antagonist (n/%)	116/21.0	77/17.0	39/39.0	2.77 (1.86–4.15)	<0.001
Digoxin (n/%)	36/6.0	16/4.0	20/20.0	4.34 (2.66–7.09)	<0.001
Amiodarone (n/%)	89/16.0	67/15.0	22/22.0	1.53 (0.95–2.46)	0.078
Acetilsalicilic acid (n/%)	172/31.0	137/30.0	35/35.0	1.23 (0.82–1.86)	0.317
Warfarin (n/%)	98/18.0	69/15.0	29/29.0	2.02 (1.31–3.11)	0.001
Statins (n/%)	197/35.0	164/36.0	33/33.0	0.87 (0.57–1.32)	0.515

CI confidence interval; NYHA New York Heart Association; AF atrial fibrillation; AV atrioventricular; NSVT nonsustained ventricular tachycardia; VP venticular pacing; LA left atrium; LVEF left ventricular ejection fraction; LVEDD left ventricular end-diastolic diameter; LVESD left ventricular end-systolic diameter; RV right ventricle; ACEi angiotensin-converting enzyme inhibitors; ARB angiotensin II receptor blocker

During the mean time of follow-up of 3.7±1.5 years, 100 patients (18.0%) died. Thirty deaths (30.0%) were due to progressive heart failure and 26 were sudden (26.0%). Eighteen patients died from non-cardiovascular causes, 11 from other non-cardiovascular causes and the cause of death could not be determined in 15 patients.

### Variable selection

**Fig 1:**
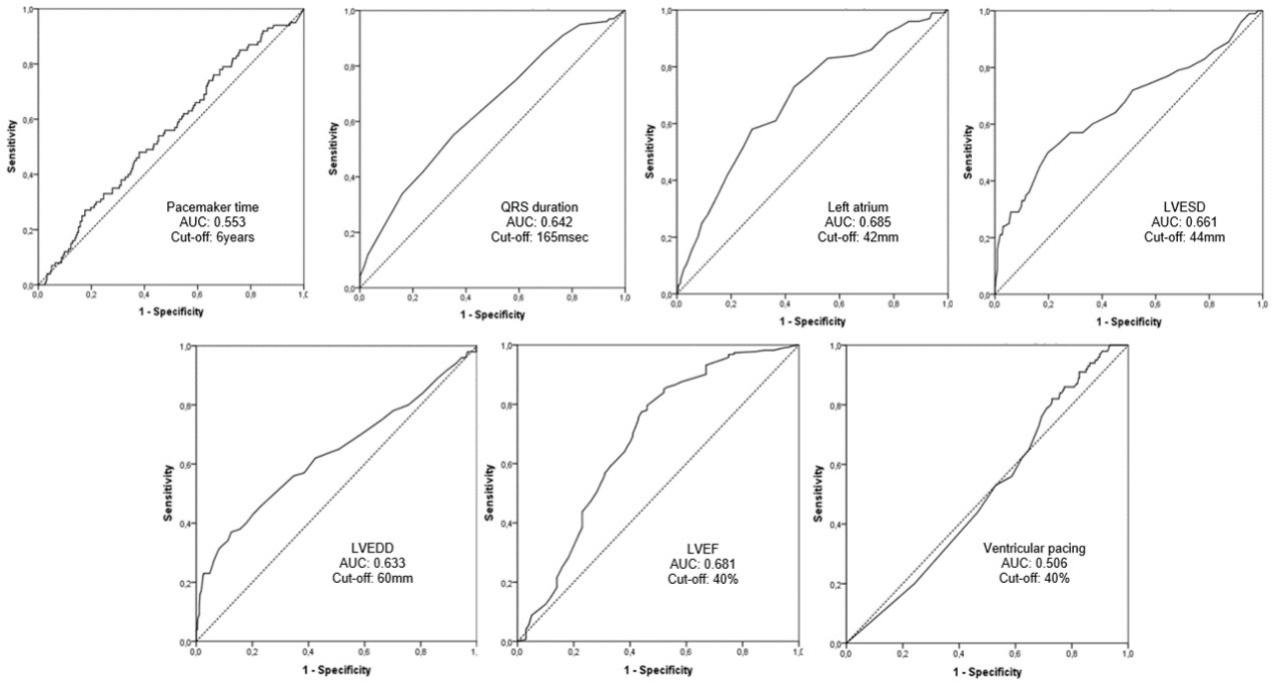
Area under the curve (AUC) for continuous variables. LVEDD left ventricular end-diastolic diameter; LVESD left ventricular end-systolic diameter; LVEF left ventricular ejection fraction.

Continuous variables that yield *P*<0.20 in the univariate analysis were evaluated by means of ROC curve in order to determine a cutoff value. (Fig 1). All these variables yield *P*<0.20 when categorized and were included in the multivariate analysis: QRS duration, left atrium diameter, left ventricular diastolic diameter, left ventricular systolic diameter, left ventricular ejection fraction, ventricular pacing and pacemaker time. Categorical variables that yield *P*<0.20 in the univariate analysis were also included in the multivariate analysis: gender, NYHA functional class, renal disease, atrial fibrillation, syncope, cardiomegaly on X-ray, NSVT at pacemaker and right ventricular dysfunction.

Six predictors of death were identified in the multivariate analysis: right ventricular dysfunction (HR 2.24; 95%CI 1.41–3.53; *P* = 0.001); heart failure functional class III or IV (HR 2.16; 95%CI 1.16–4.00; *P* = 0.014); renal disease (HR 2.14; 95%CI 1.24–3.68; *P* = 0.006); left ventricular end-systolic diameter > 44mm (HR 1.97; 95%CI 1.26–3.05; *P* = 0.003); atrial fibrillation (HR 1.94; 95%CI 1.25–2.99; *P* = 0.003) and cardiomegaly on X-ray (HR 1.87; 95%CI 1.10–3.17; *P* = 0.020).

The likelihood chi-square statistic was calculated to determine the individual contribution of each covariate to the model.

### Model performance and validation

**Fig 2:**
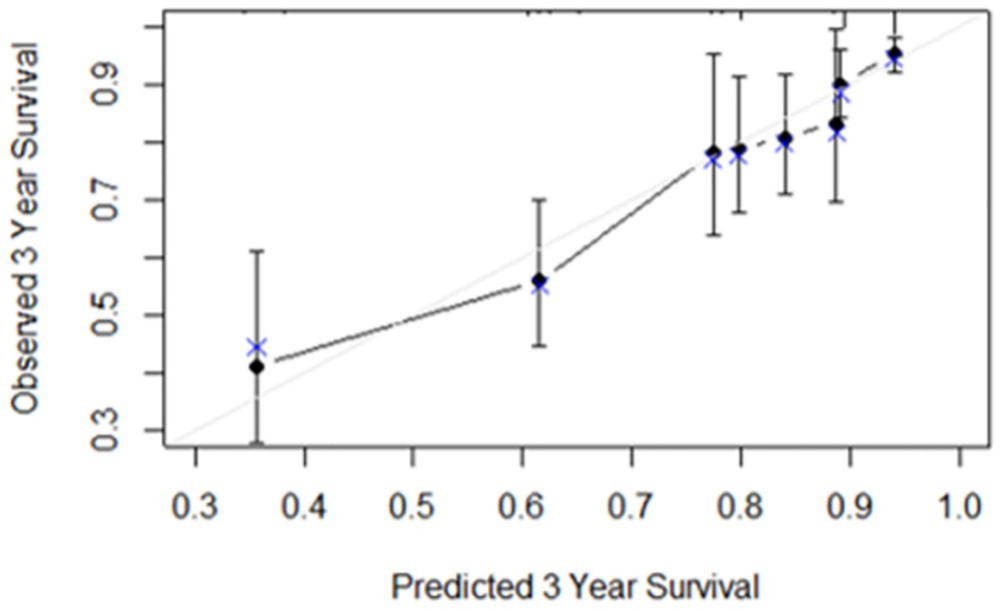
Calibration plot showing the agreement between predicted (x axis) and observed (y axis) 3-year risk of the primary outcome. Dark circles represent binned Kaplan—Meier estimates with 95% confidence intervals. Blue X represents the observed survival.

The optimism-corrected C-statistic of the predictive model was 0.751 (95% CI 0.696–0.806). Internal validation with bootstrapping revealed a calibration slope of 0.946 (95% CI 0.920–0.961), reflecting a small degree of over-optimism and C-statistic of 0.746 (95% CI 0.692–0.785). Fig 2 presents a graphical representation of calibration, showing good overall agreement between the predicted and observed 3-year risk.

### Score

To calculate the risk score, the β-coefficient of the six prognostic variables was multiplied by 20, yielding 16 points for right ventricular dysfunction, 15 points for renal disease and NYHA HF functional class III/IV, 14 points for LVESD>44mm and 13 points for cardiomegaly on x-ray and AF (Table 2).

**Table 2:** Multivariate analysis and score based on coefficient.

	Coefficient	Hazard ratio (95%CI)	*P*	Score
RV dysfunction	0.80	2.24 (1.41–3.53)	0.001	16
NYHA III/IV	0.77	2.16 (1.16–4.00)	0.014	15
Renal disease	0.76	2.14 (1.24–3.68)	0.006	15
LVESD>44mm	0.67	1.97 (1.26–3.05)	0.003	14
Atrial fibrillation	0.66	1.94 (1.25–2.99)	0.003	13
Cardiomegaly on X-ray	0.62	1.87 (1.10–3.17)	0.020	13

CI confidence interval; NYHA New York Heart Association; LVESD left ventricular end-systolic diameter; RV right ventricle

**Fig 3:**
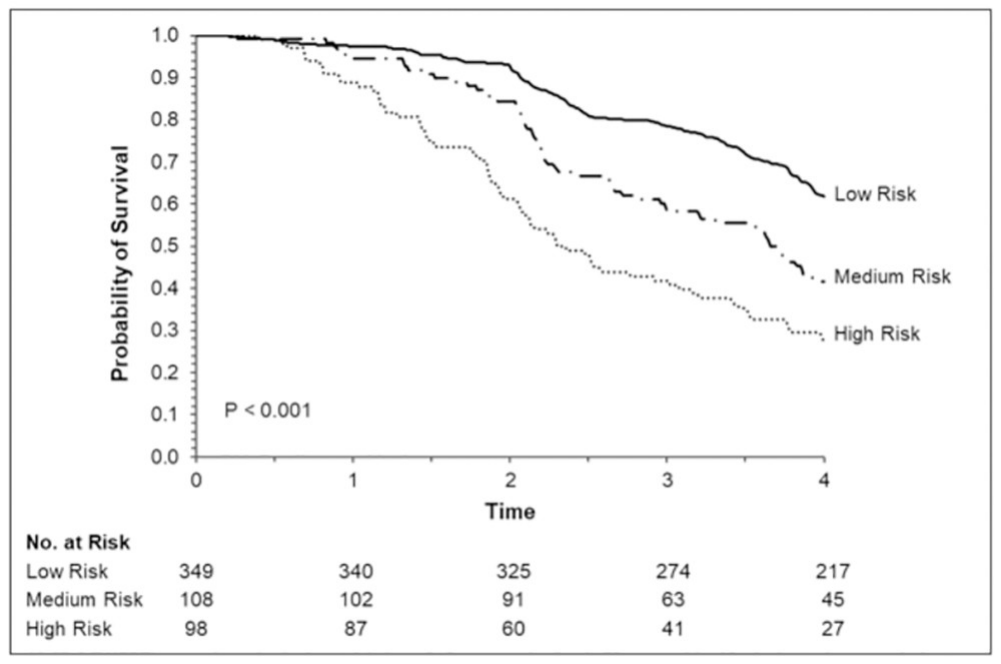
Survival estimated by the Kaplan—Meier method among the groups and Log-Rank test.

Applying the risk score to the total population, the median score was 13 (minimum value = 0, maximum value = 86, interquartile range 0–27). The increase in the score determined higher probability of death. Patients were divided into three categories according to the total score and the following risk groups were identified: 0–20, 21–30 and > 31 points. The observed rates of death were 8.0, 20.4 and 51.0, respectively (Table 3). Survival estimated by the Kaplan—Meier method among the groups was shown in Fig 3.

**Table 3:** Mortality rate according to score.

	Survivors (n = 455)	Nonsurvivors (n = 100)	Mortality rate
Low (0–20)	321 (71%)	28 (28%)	8.0%
Medium (21–30)	86 (19%)	22 (22%)	20.4%
High (30–86)	48 (10%)	50 (50%)	51.0%

## Discussion

This study addressed the mortality rate and its predictors in a large population of Chagas disease patients requiring pacemaker. We also developed a clinical score system that predicts mortality in CCC patients with pacemaker.

In the last decades many variables were studied in order to identify the predictors of poor prognosis in CCC patients. The variables identified were different among the studies, which is explained by the heterogeneity of the disease, heterogeneity of the patients included (comorbidities, medication), variables included in the multivariable models and the mean length of follow-up [14]. All of these studies contribute to a better understand of this neglected disease and our study complemented the research in this field.

Three of the six predictors of death (right ventricular dysfunction, LVESD>44mm and cardiomegaly on X-ray) are related to heart structural changes caused by Chagas disease. Interestingly, although ejection fraction was associated to death as a continuous variables or <40%, it was not an independent predictor.

AF is a well-recognized factor associated with worse prognosis, independent of structural heart disease and its prevalence increases with deterioration of ventricular function [15]. In patients with CCC with and without MPD, the prevalence of AF ranges from 3.1 to 10.4% [7,11,16] and from 9.0 and 10.0% [17], respectively. We reported a higher rate of AF (108 patients, 19.4%) and proved its relation to death, increasing the risk almost twice.

Ventricular arrhythmias are a classical risk factor associated with worse prognosis. The presence of fibrosis, dysautonomia and persistent cardiac inflammation could contribute to the risk of SCD offering a substrate for ventricular tachycardia [18]. Two studies including CCC patients found that NSVT [11] and non-specified ventricular tachycardia at stress testing or 24hour Holter [12] were predictor of death. However, in our study, NSVT was not even associated with death. This can be explained by the different methodology used to record this arrhythmia considering that pacemaker users are under 24hours rhythm monitoring and the retrieval of this information is easily obtained. Probably the other studies reported NSVT in patients with higher burden of this arrhythmia, that was properly registered by periodic exams.

The score demonstrated good ability to predict the risk of death using variables that are easily used in clinical settings. The bootstrap re-sampling technique ruled out any ‘over-optimism’ in the predictive discrimination.

The observed annual mortality rate (4.9%) was higher than the rates from the most recent studies including non-pacemaker CCC patients, which reported an annual mortality rate ranging from 1.1 to 4.0% (11,12,19). However, we must mention that our population was older, had LVEF slightly lower and higher rates of AF and cardiomegaly.

The annual rate of sudden death in our study (1.2%) was lower than other cohorts, ranging from 2.1 to 2.5% per year [11,19]. We might imagine that sudden death was less frequent in CCC patients with pacemaker since one of the mechanisms of sudden death, bradyarrhythmias, is almost impossible in the case of normal functioning devices. On the other hand, cardiac pacing in areas of fibrosis may trigger ventricular arrhythmias as already shown in studies with CRT [20].

However, our population had a higher annual mortality rate due to heart failure compared with the same aforementioned cohorts, 1.4% versus 0.6–0.7% [11,19]. It is a hard task to elucidate if the poor prognosis of CCC patients with pacemaker is related to the deleterious effect of the pacemaker, causing ventricular dyssynchrony, or an intrinsic higher risk of CCC patients with an advanced compromise of the conduction system, which can be an expression of larger areas of myocardial fibrosis. Moreover, no variable related to cardiac pacing was associated to death.

For CCC patients, our group found in a cohort of 116 CCC patients submitted to ICD for secondary prevention, that a low rate of cumulative right ventricular pacing was a predictor of better survival [10]. However, we found no association between the right ventricular pacing burden and mortality, suggesting that the lower impact of the induced LBBB could be related to the better LVEF, since the negative impact of pacemaker in patients without ventricular dysfunction is still unknown.

Considering the hypothesis that CCC patients requiring pacemaker present a higher risk of death, there is only one study addressing this point. Bestetti et al compared the survival at 1, 2 and 3 years in 110 CCC patients, 52% with pacemaker. CCC with and without pacemaker presented a survival rate of 68%, 53% and 50% versus 94%, 86% and 67%, respectively. Logistic regression analysis identified pacemaker and left ventricular dysfunction as predictors of mortality [21].

Previous published scores to predict death in CCC patients not included [11,12] or included a small number of pacemaker users, only 64 out of 1551 patients included [22]. Although in this study pacemaker use was associated to death, the authors preferred not to include this variable, as well as AF in the multivariate analysis, due to low number of patients presenting these conditions.

In 2018 we published the first results of this cohort [23]. That time we had included 396 patients and followed them for 1.9 years. We observed different predictors of death (advanced HF functional class, renal disease, QRS ≥150ms, left atrial enlargement and LVEF ≤43%) which is probably explained due to a sample increase of 40% and longer follow-up.

The limitations of our study should be pointed out. The pacemaker time was long and the patients vary greatly in terms of pacemaker time. The extended confidence interval of advanced HF functional class is related to the low prevalence of this condition, reducing the precision of the estimated risk. We evaluated the right ventricle function subjectively as good, moderate or poor, instead of a numerical method. We were unable to identify the site of the endocardial pacing, the ventricular arrhythmia burden and the cause of death in 15% of the cases. Although the bootstrap technique determined adequate internal validity, the external validity is required.

In conclusion, we developed a risk score for death in CCC patients with pacemaker, based on six predictors that can be easily applied in clinical practice Patients with these predictors must be followed closely in order to receive more aggressive therapies. If CRT or ICD will change the prognosis of these patients, remains to be elucidated.

## Supporting information

S1 Data Pacinchagas data. (XLSX)
