# Correction: Mortality risk score for patients with Chagas cardiomyopathy and pacemaker

**DOI:** 10.1371/journal.pntd.0012928

**Published:** 2025-03-12

**Authors:** Giselle de Lima Peixoto, Sérgio Freitas de Siqueira, Silvana Angelina D’Orio Nishioka, Anísio Alexandre Andrade Pedrosa, Ricardo Alkmim Teixeira, Roberto Costa, Martino Martinelli Filho

There is an error in affiliation 1 for the authors. The correct affiliation 1 is: Unidade Clínica de Estimulação Cardíaca Artificial, Instituto do Coração, Hospital das Clínicas HCFMUSP, Faculdade de Medicina, Universidade de São Paulo, São Paulo, Brazil.

## References

[1] de Lima Peixoto G, de Siqueira SF, Nishioka SAD, Pedrosa AAA, Teixeira RA, Costa R, et al. Mortality risk score for patients with Chagas cardiomyopathy and pacemaker. PLoS Negl Trop Dis. 2024;18(5):e0012114. doi: 10.1371/journal.pntd.0012114 38723058 PMC11164388

